# Dysglycemia induces abnormal circadian blood pressure variability

**DOI:** 10.1186/1475-2840-10-104

**Published:** 2011-11-22

**Authors:** Sivarajan Kumarasamy, Kathirvel Gopalakrishnan, Dong Hyun Kim, Nader G Abraham, William D Johnson, Bina Joe, Alok K Gupta

**Affiliations:** 1 Clinical and Population Research, Pennington Biomedical Research Center, Louisiana State University System, 6400 Perkins Road, Baton Rouge, LA 70808, USA; 2 Physiological Genomics Laboratory, 336, Block Health Science Building, Department of Physiology and Pharmacology, University of Toledo College of Medicine and Life Sciences, 3000 Arlington Avenue, Toledo, OH 43614, USA

**Keywords:** caloric excess, adipose tissue dysfunction, insulin resistance, renin-aldosterone-angiotensin system, circadian blood pressure variability, adipokines, leptin, adiponectin, pro-inflammatory cytokines, MCP-1, TNF-α, early CVD risk

## Abstract

**Background:**

Prediabetes (PreDM) in asymptomatic adults is associated with abnormal circadian blood pressure variability (abnormal CBPV).

**Hypothesis:**

Systemic inflammation and glycemia influence circadian blood pressure variability.

**Methods:**

Dahl salt-sensitive (S) rats (n = 19) after weaning were fed either an American (AD) or a standard (SD) diet. The AD (high-glycemic-index, high-fat) simulated customary human diet, provided daily overabundant calories which over time lead to body weight gain. The SD (low-glycemic-index, low-fat) mirrored desirable balanced human diet for maintaining body weight. Body weight and serum concentrations for fasting glucose (FG), adipokines (leptin and adiponectin), and proinflammatory cytokines [monocyte chemoattractant protein-1 (MCP-1) and tumor necrosis factor-α (TNF-α)] were measured. Rats were surgically implanted with C40 transmitters and blood pressure (BP-both systolic; SBP and diastolic; DBP) and heart rate (HR) were recorded by telemetry every 5 minutes during both sleep (day) and active (night) periods. Pulse pressure (PP) was calculated (PP = SBP-DBP).

**Results:**

[mean(SEM)]: The AD fed group displayed significant increase in body weight (after 90 days; p < 0.01). Fasting glucose, adipokine (leptin and adiponectin) concentrations significantly increased (at 90 and 172 days; all p < 0.05), along with a trend for increased concentrations of systemic pro-inflammatory cytokines (MCP-1 and TNF-α) on day 90. The AD fed group, with significantly higher FG, also exhibited significantly elevated circadian (24-hour) overall mean SBP, DBP, PP and HR (all p < 0.05).

**Conclusion:**

These data validate our stated hypothesis that systemic inflammation and glycemia influence circadian blood pressure variability. This study, for the first time, demonstrates a cause and effect relationship between caloric excess, enhanced systemic inflammation, dysglycemia, loss of blood pressure control and abnormal CBPV. Our results provide the fundamental basis for examining the relationship between dysglycemia and perturbation of the underlying mechanisms (adipose tissue dysfunction induced local and systemic inflammation, insulin resistance and alteration of adipose tissue precursors for the renin-aldosterone-angiotensin system) which generate abnormal CBPV.

## Background

Obesity is universally associated with excess adipose tissue distribution [1,2]. Excess adipose tissue in ectopic locations: especially in the visceral compartment, liver and muscle, is dysfunctional. The secreted adipokines (including cytokines and chemokines) mediating auto, para and endocrine actions, alter the dynamic homeostatic milieu to enhance systemic inflammation, which results in dysglycemia, dyslipidemia, and/or a loss of control of blood pressure [3-6]. These early changes are clinically manifest as prediabetes and prehypertension, in advance of the chronic changes that subsequently (at times10-15 years later) lead to a diagnosis of diabetes mellitus and hypertension [4,7]. The early increase in pro-inflammatory and pro-coagulant factors, reactive oxygen species, dysglycemia, dyslipidemia, and loss of blood pressure control, are functionally detectable as abnormal circadian blood pressure variability and endothelial dysfunction [8].

Asymptomatic overweight adults with prediabetes, when compared to overweight adults with normal glucose, were observed to have abnormal circadian blood pressure variability [9]. In a separate study involving asymptomatic obese adults, only those with prediabetes and a highly potentiated systemic inflammation, (in comparison to a matched obese group with normal fasting glucose and marginally elevated systemic inflammation), displayed not only abnormal circadian blood pressure variability, but also demonstrated endothelial dysfunction [8]. Prediabetes and prehypertension in otherwise healthy adults singly, or together (co-existing prediabetes and prehypertension) place the individual on a pathway with potential for accelerated cardiovascular adverse events, including sudden death [10-13].

These results led us to formulate our overall hypothesis that short term caloric excess results in adipose tissue deposition. The excess ectopic adipose tissue alters its adipokine secretion menu tipping the pro-inflammatory and anti-inflammatory balance in favor of inflammation (leading to altered glycemia and lipidemia) and potentiating the elements of the renin-angiotensin-aldosterone system (resulting in a loss of blood pressure control). This latent high cardiovascular risk is clinically manifest as prediabetes and/or prehypertension and functionally as abnormal circadian blood pressure variability and/or endothelial dysfunction.

Using an animal model with a permissive genetic background we tested the hypothesis that systemic inflammation and glycemia influence circadian blood pressure variability.

## Subjects & Methods

### Ethics Statement

All animal procedures and protocols used in this report were approved by the University of Toledo Health Science Campus Institutional Animal Care and Use Committee (IACUC protocol numbers 105276, 104573 and 104045).

### Animals

The animal experiments were performed at the Physiological Genomics Laboratory, Department of Physiology and Pharmacology, University of Toledo College of Medicine. Dahl salt-sensitive (S) rats (n = 19) from the colony maintained at the University of Toledo College of Medicine and Life Sciences, Toledo, Ohio were used. These rats with a genetically permissive background allowed us to simulate the changes that occur with the feeding of an American diet and compare them to a standard diet. We were able to demonstrate the early effects of caloric excess, the metabolic changes and their physiologic consequences. The rats were weaned at 30 days and fed either an American diet (AD) or a standard diet (SD) for the duration of the study (six months).

### Diets

American diet (AD; Teklad Custom TD.08811; high-glycemic-index, high-fat) was designed to simulate the customary every day human diet which due to its overabundant calories on a daily basis, over time, leads to body weight gain. The standard diet (SD; TD.08810; low-glycemic-index, low-fat) was designed to mirror the desirable balanced human diet which would sustain growth and maintain body weight.

### Body weight

Individual body weight of rats in the AD and SD fed groups was measured at 90,120,135, 160 and 172 days of age. A group mean(SEM) body weight was reported at these time points.

### Biochemical analysis

#### Fasting blood glucose

The blood glucose concentrations were measured using a commercially available 'One Touch Ultra Smart' kit according to the manufacturer's instructions. Rats were fasted overnight and individual blood samples were collected at age 90 and 172 days. The group (AD or SD) mean(SEM) fasting glucose were calculated from individual measurements and were reported.

#### Cytokine analyses

All the cytokine analysis (leptin, adiponectin, monocyte chemoattractant protein-1 and tumor necrosis factor) were performed using a Multiplex Adipokine/Adipocyte Panels (Assay Gate, Inc, Ijamsville, MD, USA) according to the manufacturer's instructions. The blood samples were collected for cytokine analysis from individual rats included in the AD and SD fed groups. The individual mean of duplicate measures from each individual rat were used to report the group mean(SEM) concentration. At the end of study all the animals were euthanized. Individual body weights, heart and kidney weights were recorded. A group mean(SEM) heart and kidney weight was reported as a surrogate measure for ectopic fat deposition.

### Blood pressure measurement

Systolic blood pressure, diastolic blood pressure and heart rate were obtained by using a telemetry system (Data Sciences International, St. Paul, MN) as previously detailed [14]. Pulse pressure was calculated (PP = SBP-DBP). In brief, 30 day old rats were weaned and fed either an AD or a SD. When the rats were 80 days old, C40 telemetry probes were surgically implanted through their femoral arteries and advanced into their lower abdominal aorta. The rats were then allowed to recover from surgery for 4 days, before the transmitters were switched on for recording BP and HR. All statistical analyses were conducted as previously reported [14].

### Statistical Analysis

Data were analyzed using the student's t-test to assess statistical significance. Data are presented as the mean(standard error of mean). Statistical significance was reported as p ≤ 0.001, ≤ 0.01 or ≤ 0.05.

## Results

### Body weight

**Figure 1:**
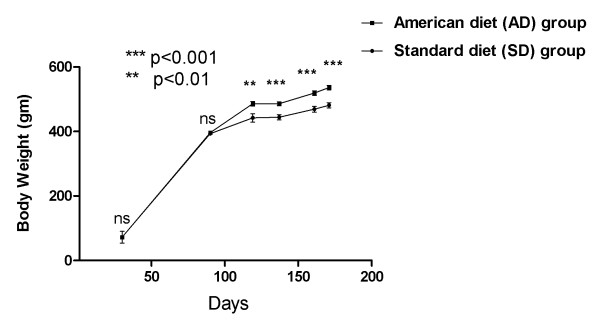
**Body weight in AD and SD fed rats: Mean(SEM) change with time**.

Dahl salt-sensitive (S) rats (n = 19) were weaned and fed either an American diet (AD: designed to promote weight gain; n = 10) or a standard diet (SD: designed to maintain normal growth weight; n = 9) for six months. There were no significant differences in the mean body weight of the group fed with the AD in comparison with the group fed SD at 90 days. A significant differential increase in mean body weight (p < 0.01 at 120 days, p < 0.001 at 135 days, p < 0.001 at 160 days) then ensued in the AD fed group, which persisted until the end of the experiment (172 days; p < 0.001). Figure 1 details the body weight changes.

### Glycemia

**Figure 2:**
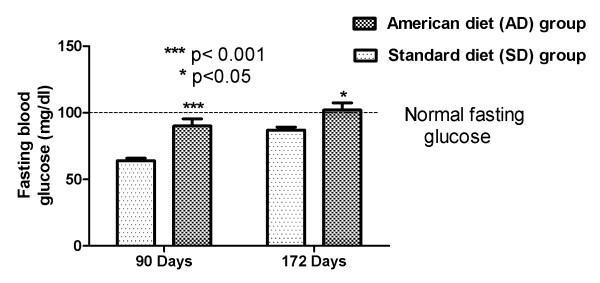
**Glycemia in AD and SD fed rats: Mean(SEM) fasting blood glucose change**.

Fasting glucose assessments were obtained with a One Touch Ultra Smart kit at two different time points: 90 and 172 days of age. At the age of 90 days the AD fed group displayed significantly increased fasting blood glucose (FBG) 90.1(5.38) mg/dL compared to SD fed group 63.7(1.98) mg/dL, (p < 0.001). The AD fed group at the age of 172 days exhibited an even higher mean FBG 102(5.4) mg/dL (compared to SD fed group 87(2.33) mg/dL, p < 0.05). Fasting serum glucose concentrations above 100 mg/dL in humans are associated with a diagnosis of prediabetes, a recognized pre-disease state with high covert risk of cardiovascular disease, and the potential for conversion to diabetes mellitus. Figure 2 demonstrates fasting blood glucose in the AD and SD fed rats.

### Adipokines

**Figure 3:**
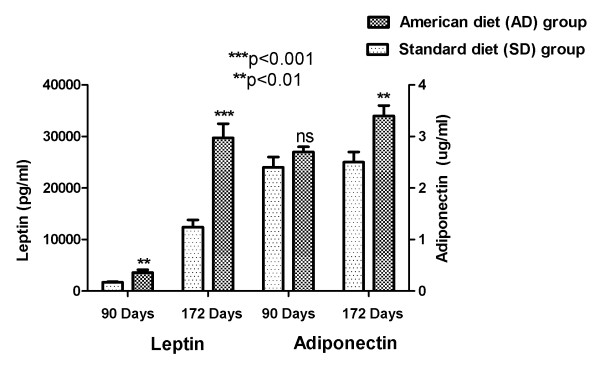
**Adipokines in AD and SD fed rats: Mean(SEM) Leptin and Adiponectin change**.

All the cytokine analysis (leptin, adiponectin, monocyte chemoattractant protein-1 and tumor necrosis factor) were performed using a Multiplex Adipokine/Adipocyte Panels (Assay Gate, Inc, Ijamsville, MD, USA). A significant increase in serum leptin (an index of AD induced escalation in adiposity) in the group fed with AD (in comparison to the group fed SD) was seen at both 90 (p < 0.01) and 172 days of age (p < 0.001). This figure also demonstrates the beginning of an increase in serum adiponectin with AD at three months, which attained significance (p < 0.01) at the end of the study. This is indicative of a functional adipose tissue increasing the secretion of its major adopokine, adiponectin with the increase in adipose tissue mass. Figure 3 depicts the adipocyte secreted adipokines: leptin and adiponectin.

### Systemic inflammation

**Figure 4:**
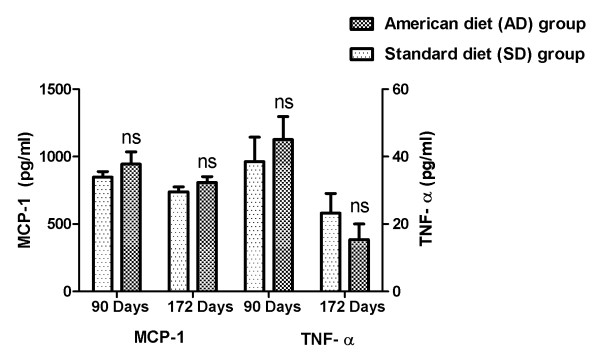
**Pro-inflammatory cytokines in AD and SD fed rats: Mean(SEM) MCP-1 and TNF-α change**.

An initial increase in the systemic pro-inflammatory cytokines, MCP-1 and TNF-α in the AD fed group (in comparison to the group fed SD) at three months, subsequently declined at end of the study. Although the means from the groups did not reach significance at either time point, this increase is indicative of ectopic adipose tissue deposition, adipose tissue dysfunction and an augmented systemic pro-inflammatory secretory activity. Figure 4 details the proinflammatory cytokines: MCP-1 and TNF-α

### Organ mass

**Figure 5:**
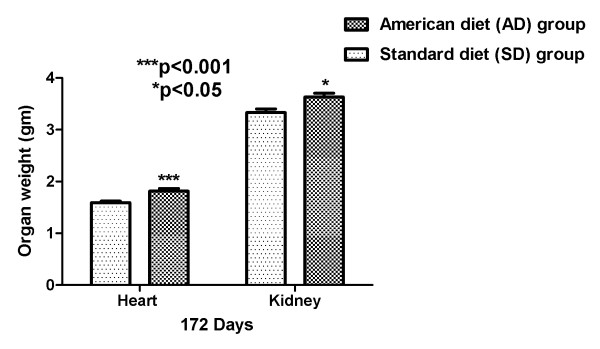
**Organ weights in AD and SD fed rats: Heart and Kidney**.

After being euthanized at the end of the experiment, total body and organ (heart and kidney) weights were obtained. AD fed group showed significantly (p < 0.001 and p < 0.05) increased heart and kidney weights, when compared with SD fed rats. The higher organ weights denote ectopic fat deposition and the likely consequent organ dysfunction. Figure 5 shows the heart and kidney weight of the AD and SD fed rats.

### Circadian blood pressure variability

The blood pressure was recorded by radio-telemetry every five minutes for several days and nights at two different time points (TP-1: age 85-94 days and TP-2: 106-116 days). The daily average for each day for TP-1 (days 85-94) and TP-2 (days 106-116) in each AD fed rat was significantly higher (p < 0.001) than each SD fed rat. The overall mean(SEM) of 2318 and 2782 individual measures obtained during the TP-1 and TP-2 from each member rat in the AD and the SD fed groups, respectively, were used to report the results below.

The American diet fed rats exhibited significantly increased overall mean systolic and diastolic blood pressures compared with standard diet fed for all the time points: TP-1 SBP 165.05(0.17) vs. 148.23(0.14) mm Hg, p = 0.02; DBP 124.89(0.14) vs. 111.87(0.13) mm Hg, p = 0.02; TP-2 SBP 174.78(0.15) vs. 153.14(0.12) mm Hg, p = 0.01; DBP 132.34(0.13) vs. 116.63(0.11) mm Hg, p = 0.03.

The mean night time (active period) systolic and diastolic pressures in the AD fed group were significantly higher when compared with SD fed group: TP-1 SBP 168.91(1.07) vs. 151.40(0.96) mm Hg, p = 0.02; DBP 128.06(0.78) vs. 114.78(0.86), p = 0.03; TP-2 SBP 173.86(1.69) vs. 152.58(1.06) mm Hg, p = 0.02; DBP 131.56(1.40) vs. 116.45(0.76) mm Hg, p = 0.04.

The mean day time (sleep period) systolic and diastolic pressures in the AD fed group were significantly higher when compared with SD fed group: TP-1 SBP 160.94(1.01) vs. 145.23(0.95) mm Hg, p = 0.02; DBP 121.57(0.89) vs. 109.1(0.91) mm Hg, p = 0.03; T2 SBP 175.42(0.66) vs. 153.57(0.38) mm Hg, p = 0.02; DBP 132.83(0.54) vs. 116.62(0.35) mm Hg, p = 0.03.

The night time increase in mean SBP (17.51 mm Hg) and DBP (13.28 mm Hg) in AD fed rats during TP-1 not only persisted, but was higher during TP-2 SBP (21.28 mm Hg) DBP(15.11 mm Hg).

### Systemic inflammation and fasting glucose influence circadian blood pressure variability

**Figure 6:**
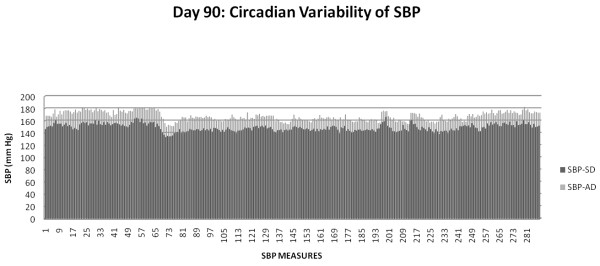
**SBP in AD and SD fed group: Day 90**.

**Figure 7:**
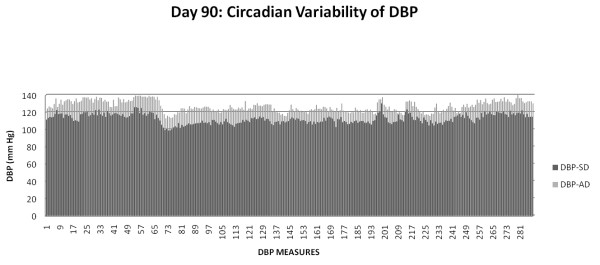
**DBP in AD and SD fed group: Day 90**.

**Figure 8:**
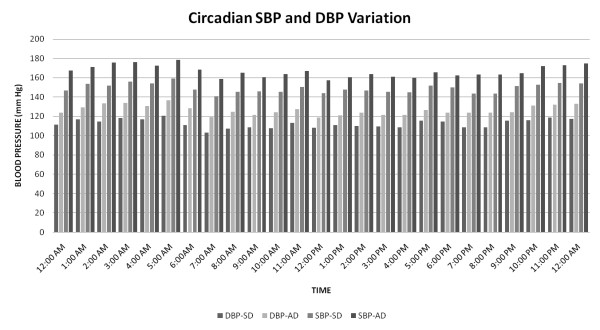
**Hourly SBP and DBP: Day 90**.

Figures 6, 7 and 8 detail circadian blood pressure on day 90 when enhanced systemic inflammation and increased fasting blood glucose was demonstrated in the AD fed rats.

Figure 6 compares SBP in the AD fed group with the SD fed group during one 24 hour period (day 90). Each one of the 288 SBP measurements in the AD fed group was higher than the SD fed group over the course of day 90, p = 0.02. The mean MCP-1 (944.6 pg/ml vs. 848 pg/ml) and TNF-α (45.1 vs. 38.5 pg/ml) were higher in the AD fed group when compared with the SD fed group on day 90, but did not reach significance (as shown in Figure 4). A fasting glucose measure performed on day 90 showed significantly higher concentrations in the AD fed group 90.1(5.38) vs. 63.7(1.98) mg/dL in the SD fed group, (p < 0.001) (as shown in Figure 2).

Figure 7 depicts the DBP in the AD fed group with the SD fed group during one 24 hour period (day 90). Each one of the 288 DBP measurements in the AD fed group was higher than the SD fed group over the course of day 90, (p = 0.02), the time period when the fasting glucose was also significantly (p < 0.001) higher.

Figure 8 is a graphic demonstration of the mean hourly systolic and diastolic blood pressures every hour over the course of day 90. Every one of the 24 measures of the systolic and diastolic blood pressure in the AD fed group was significantly higher, p < 0.05 when compared to the SD fed group.

### American diet promotes abnormal circadian blood pressure variability

**Figure 9:**
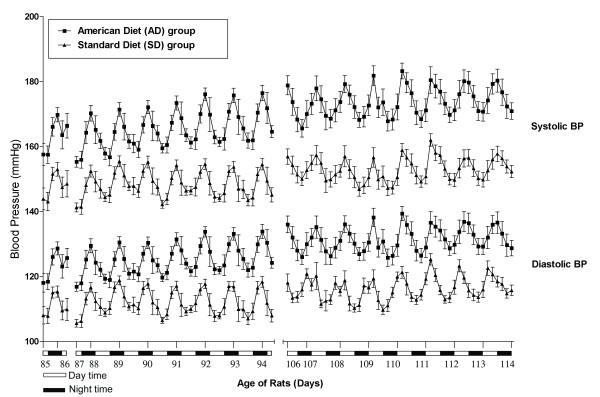
**Blood Pressure in AD and SD fed rats: Circadian systolic and diastolic blood pressure variability**.

**Figure 10:**
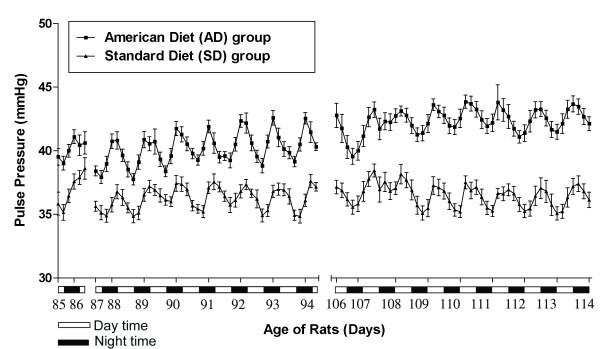
**Pulse pressure in AD and SD fed rats: Circadian pulse pressure variability**.

**Figure 11:**
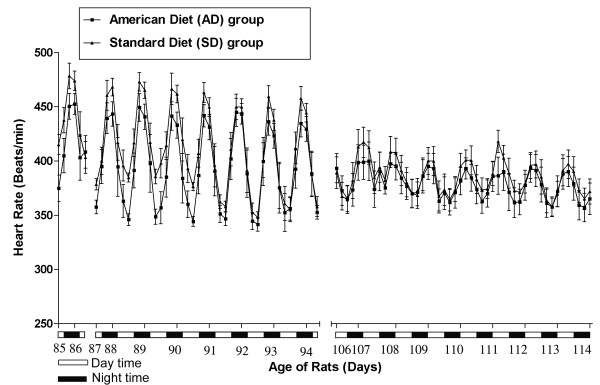
**Heart rate in AD and SD fed rats: Circadian heart rate variability**.

Figures 9, 10 and 11 show the aggregate day and night 4 hour means(SEM) of SBP, DBP, PP and HR in the AD fed group compared SD fed group over the course of TP-1 and TP-2.

Figure 9 shows a graphic depiction of the significantly higher (p < 0.001) mean(SEM) day and night time systolic and diastolic blood pressures in the AD and SD fed rats, throughout the course of TP-1 and TP-2.

Figure 10 graphically depicts mean(SEM) pulse pressure in the AD fed and SD fed rats. The pulse pressure (calculated from the systolic and the diastolic blood pressure) of AD fed rats over the course of TP-1 and TP-2 was significantly (p < 0.001) higher than that of SD fed rats.

Figure 11 displays the mean(SEM) heart rate in the AD fed and SD fed rats. The AD fed rats show a significantly (p < 0.05) increased heart rate when compared with the SD fed rats over the course of TP-1 and TP-2.

## Discussion

This study illustrates the early relationship between caloric over-loading (modeled after the usual American diet), an increased systemic inflammation, fasting serum glucose and loss of blood pressure control. The biphasic response (possibly T-cell mediated) of the pro-inflammatory cytokines (MCP-1, and TNF-α) is evident with the increased serum concentrations at three months, followed by a decrease at six months (Figure 4). This change was accompanied with a significant linear increase with time in serum adipokines, leptin (indicative of escalating adiposity) and adiponectin (reflective of an increased functional adipose tissue mass) (Figure 3). We were able to demonstrate the early onset of insulin resistance by showing a differentially significant increase in fasting blood glucose at three months in rats fed with AD diet, without a differential increase in body weight (Figure 2). This significant increase in fasting glucose, driven by the dysfunctional, excess adipose tissue in ectopic locations, was also accompanied by abnormal circadian blood pressure variability. Prediabetes in healthy human subjects has been demonstrated to be positively associated with an increased arterial stiffness: independent of the confounding variables (including age, gender, BMI, blood pressure, resting heart rate, hs-CRP, lipid profile, and behavioral habits) [15].

In conjunction with the persistently high and escalating fasting glucose, at both three and six months, (without and with weight gain, respectively), we elucidate abnormalities of circadian blood pressure variability. We demonstrate an increase in each of the 2318 and 2782 individual blood pressure measures, obtained during the reference days (TP-1: 85-89 and TP-2: 106-114 days, respectively) from each American diet fed rat, compared with a standard diet fed rat. We report that all the isolated 288 blood pressure measures on day 90, during demonstrated increased systemic inflammation (Figure 4) and significantly increased fasting glucose (Figure 2), were significantly higher (Figures 6 and 7). The hourly mean SBP and DBP in presence of exacerbated systemic inflammation (TNF-α, MCP-1: Figure 4) and significantly elevated fasting glucose (Figure 2) on day 90, were all significantly higher (Figure 8). The significantly elevated mean SBP, DBP, PP and HR in the American diet fed group remained evident and persisted until the end of the experiment (four hour mean(SEM) SBP, DBP, PP and HR are shown in Figures 9, 10 and 11). The weight of the heart and the kidneys, indicative of ectopic adipose tissue deposition, was significantly higher in the group subject to caloric excess at the end of the experiment (Figure 5).

The association of obesity with diabetes mellitus and hypertension is widely recognized. Given that obesity is a low grade chronic inflammatory disease and that inflammation plays an integral part in the pathogenesis of diabetes and hypertension, it should be a simple matter to show that obesity causes inflammation, which causes insulin resistance leading to diabetes, and loss of blood pressure control, leading to hypertension. This has, however, not been the case. The interrelationships between obesity, diabetes and hypertension are, at best, complex and are not yet clearly defined. In a recently published study, although the obese with higher degree of inflammation had a higher propensity to develop hypertension over time, inflammation by itself statistically did not add to the risk of developing diabetes or hypertension [16].

Since the efforts by investigators thus far have been to elucidate the relationships between obesity, diabetes and hypertension, after the diseases had reached an advanced stage, we designed a study to illustrate the process in an early, more acute setting. We chose to elucidate the relationship between excess caloric intake, systemic inflammation, dysglycemia (prediabetes), loss of blood pressure control (prehypertension) and the alteration in circadian blood pressure variability.

The conventional American diet (high-glycemic-index, high fat) fed to rats after weaning, would lead to both hyperplasia (increase in number) and hypertrophy (increase in size) of adipose tissue cells [17]. This would then result in altered secretion of adipokines, chemokines and cytokines [18,19]. Elements of the renin-angiotensin-aldosterone system (RAAS), which have also been shown to be secreted by the adipose tissue, would also be potentiated [20]. The resulting imbalance between the pro-inflammatory and anti-inflammatory adopokine secretions, and the augmentation of the RAAS, would lead to dysglycemia, loss of blood pressure control and an alteration of the normal circadian blood pressure variability.

The earliest change in a hypertrophic and hyperplastic adipose tissue is the infiltration of inflammatory cells. While a significant increase in the number of normal resident adipose tissue macrophages is widely known to ensue with obesity [21], the first adipose tissue infiltrators, as insulin resistance develops, are now known to be the T-lymphocytes [22]. These T-cells, with their activation and hyperpolarization into a pro-inflammatory T-helper 1 phenotype, appear to play a pivotal role in initiating (as early as five weeks in high fat fed mice) [23], and perpetuating adipose tissue inflammation [24-26]. It is plausible that the early T-cell infiltration results in the observed early spike in the pro-inflammatory (increased MCP-1 and TNF-α) response. This is followed by a decline in response, before the activation and action of the infiltrated macrophages: explaining the observed biphasic response of the pro-inflammatory secretions. Adipose tissue and the infiltrated cells produce several pro-inflammatory, pro-coagulant, and acute-phase molecules in direct proportion to the degree of adiposity [21,27]. Among these molecules: adipokines are directly secreted by the adipocytes (leptin, adiponectin, visfatin, vaspin), while the other chemokines and cytokines are produced in conjunction by the stromal vascular components and the infiltrating cells (MCP-1, TNF-α, interleukin-6 (IL-6), plasminogen activator inhibitor-1 (PAI-1), nitric oxide (NO) and factor VII). These have all been implicated in the development of the co-morbidities associated with obesity [28]. It appears that the balance between the classically and alternatively activated macrophages, (MI and M2, respectively), subsequently determines the overall dominance of either the pro-inflammatory, or the anti-inflammatory adipose tissue secretory milieu [29].

*Limitations: This well designed experiment was done in genetically permissible rats to allow us to elucidate the early influence of caloric excess upon systemic inflammation and glycemia and their impact on circadian blood pressure variability. Although these results cannot be directly extrapolated to humans, the differences between the control group on usual diet and the test group fed with the American diet have validated our stated hypothesis. We were able to show that both systemic inflammation and glycemia influence circadian blood pressure variability. We were further able to demonstrate that dysglycemia induces abnormal circadian blood pressure variability*.

## Conclusions

This study, for the first time, demonstrates a cause and effect relationship between caloric excess, enhanced systemic inflammation, dysglycemia, loss of blood pressure control and abnormal circadian (24-hour) blood pressure variability.

## Competing interests

SK, KG, DHK, NGA, WDJ, BJ and AKG do not report any conflicts of interests.

## Authors' contributions

AKG conceived the study, arranged the collaboration, initiated the manuscript, edited and compiled the final version for submission. SK, KG, DHK, NGA and BJ performed the experiments and gathered the data. WDJ directed the statistical analyses and participated in editing of the manuscript. SK, WTC, and BJ participated in editing of the manuscript. All authors have read and agree for the publication of this manuscript.
